# Pharmacological intervention of curcumin via the NLRP3 inflammasome in ischemic stroke

**DOI:** 10.3389/fphar.2023.1249644

**Published:** 2023-10-17

**Authors:** Xiaoxue Du, Nashwa Amin, Linhao Xu, Benson O. A. Botchway, Bo Zhang, Marong Fang

**Affiliations:** ^1^ Translational Medicine Research Center, Key Laboratory of Clinical Cancer Pharmacology and Toxicology Research of Zhejiang Province, Affiliated Hangzhou First People’s Hospital, Zhejiang University School of Medicine, Hangzhou, China; ^2^ Institute of System Medicine, Zhejiang University School of Medicine, Hangzhou, China; ^3^ Department of Zoology, Faculty of Science, Aswan University, Aswan, Egypt; ^4^ Department of Cardiology, Affiliated Hangzhou First People’s Hospital, Zhejiang University School of Medicine, Hangzhou, China; ^5^ Department of Neurology, Children’s Hospital of Zhejiang University School of Medicine, National Clinical Research Centre for Child Health, Hangzhou, China; ^6^ Pharmacy Department, Bupa Cromwell Hospital, London, United Kingdom; ^7^ Key Laboratory of Clinical Cancer Pharmacology and Toxicology Research of Zhejiang Province, Affiliated Hangzhou First People’s Hospital, Zhejiang University School of Medicine, Hangzhou, China

**Keywords:** curcumin, ischemic stroke, NLRP3 inflammasome, inflammation, neuroprotection

## Abstract

Ischemic-induced neuronal injury arises due to low oxygen/nutrient levels and an inflammatory response that exacerbates neuronal loss. NOD-like receptor family pyrin domain-containing 3 (NLRP3) is an important regulator of inflammation after ischemic stroke, with its inhibition being involved in nerve regeneration. Curcumin, a main active ingredient in Chinese herbs, plays a positive role in neuronal repair and neuroprotection by regulating the NLRP3 signaling pathway. Nevertheless, the signaling mechanisms relating to how curcumin regulates NLRP3 inflammasome in inflammation and neural restoration following ischemic stroke are unknown. In this report, we summarize the main biological functions of the NLRP3 inflammasome along with the neuroprotective effects and underlying mechanisms of curcumin via impairment of the NLRP3 pathway in ischemic brain injury. We also discuss the role of medicinal interventions that target the NLRP3 and potential pathways, as well as possible directions for curcumin therapy to penetrate the blood–brain barrier (BBB) and hinder inflammation in ischemic stroke. This report conclusively demonstrates that curcumin has neuroprotective properties that inhibit inflammation and prevent nerve cell loss, thereby delaying the progression of ischemic brain damage.

## 1 Introduction

Cerebral ischemia/stroke (CIS) causes severe tissue hypoxia and brain disorders due to endothelial cell dysfunction, over-activated inflammation, and neuronal loss instigated by insufficient or no oxygen and glucose supply to the brain. According to the World Stroke Organization, more than 12.2 million new stroke cases are reported annually, of which 62% are ischemic strokes. This can cost up to $860 billion annually. CIS is detrimental and may result in death and disability due to risk factors such as high systolic blood pressure, high fasting plasma glucose, and smoking; these induce hypertension, impaired glucose tolerance, and abnormal lipid metabolism (Feigin et al., 2022).

Currently, the main therapeutic strategy for CIS in clinical settings is thrombolytic surgeries such as intravenous thrombolysis and mechanical clot thrombectomy to restore blood flow as quickly as possible, while anti-coagulant and vasoprotective drugs are also employed. However, less than 50% of thrombolytic surgeries globally are statistically effective, and affected individuals lose nearly 72% of their healthy lives to stroke-related disability and death (Saini et al., 2021). The main reason for these could be inflammatory storm and neuronal injury in CIS and reperfusion after surgery. Once cerebral infarcts occur, the loss of neurons aggravates neuronal injuries by downregulating processes related to neuroprotective and regenerative biomarkers and pathways (Datta et al., 2011). In ischemic-induced vascular obstruction, oxidative stress causes the excessive production of reactive oxygen species (ROS), exacerbating neuronal injury and resulting in severe deficits (Zhao et al., 2022). Furthermore, the breakdown of the blood–brain barrier (BBB) leads to calcium overload and mitochondrial dysfunction due to ionic imbalance and glutamate excitotoxicity. This augments the risk of oxidative stress, neuronal apoptosis, and inflammation (Minutoli et al., 2016; Subedi and Gaire, 2021). It is therefore not surprising that pathways relating to oxidative stress have been extensively investigated for mitigating neuronal injuries (Chen et al., 2020). Effective modulation of the immune responses may also minimize neuronal injuries.

The NLRP inflammasome is a key tripartite multi-protein composite that contains the nucleotide-binding oligomerization domain (NOD), C-terminal caspase-recruitment domain (CARD), and activated caspase-1 that regulates inflammatory response and neuronal pyroptosis in ischemic stroke (Sarmah et al., 2020). When the inflammatory cascade begins, the NOD-like receptor (NLR) receives the inflammatory and DAMPs danger signals to trigger the mutation and release of IL-18 and IL-1β, thus accelerating neuronal dysfunction and causing BBB breakdown, cerebral edema, and eventual neuronal death (Kim et al., 2016). There are more than 20 members of the NLRP inflammasome family, including NLRP1, NLRP3, NLRP4, and NLRP6. NLRP3, one of the most investigated, is found in microglia and neurons in the brain and has been linked to neurogenesis, angiogenesis, neuroinflammation, and neuronal recovery in Alzheimer’s disease (AD), Parkinson’s disease (PD), and ischemic stroke (Shen et al., 2022). NLRP3 dysfunction leads to the activation of pro-inflammatory cytokines and ROS overproduction, as well as autophagic and mitochondrial impairments (Cicolari et al., 2021; Holbrook et al., 2021; Anderson et al., 2023). In contrast, inhibited NLRP3 showed neuroprotective properties in cognitive functional recovery and vasoneuronal remodeling after ischemia (Ward et al., 2019). Therefore, the NLRP3 inflammasome may be a promising therapeutic approach for ischemic stroke.

In recent years, significant investigation of curcumin has demonstrated its anti-tumorigenic, anti-inflammatory, anti-neuronal apoptosis, and blood vessel protective features (Jiang et al., 2007; Menon and Sudheer, 2007). Curcumin is the primary component of turmeric rhizome and a well-known polyphenolic agent. Its poor bioavailability and solubility have resulted in its limited clinical employment. However, studies on the nanoparticles and liposomes of curcumin in drug delivery systems have attempted to significantly improve its solubility, stability, and safety (Mahmood et al., 2015). Based on its pathomechanism, curcumin has been demonstrated to alleviate various acute and chronic neuronal disorders including depression, ischemic stroke, AD, brain injury, and spinal cord injury by regulating neuroprotective pathways and downregulating the inflammatory storm, thereby enhancing neurological functions (Bhat et al., 2019; Ramaholimihaso et al., 2020; Saleh et al., 2022; Gu et al., 2023). In ischemic stroke, curcumin has upregulated the expression of synaptic remodeling proteins, decreased brain infarct size, and enhanced BBB permeability (Wu et al., 2021), as well as the release of neurotransmitters and neurotrophic substances by curcumin-upregulated signal pathways for neuronal regeneration (Lan et al., 2018). Improving ischemic brain curcumin-inhibited cell apoptosis, ROS-induced inflammation, and modulated mitochondrial functions are key factors in triggering the NLRP3 inflammasome (Subedi and Gaire, 2021). When NLRP3 inflammasome activation was impaired by curcumin, mitigated inflammation and improved neuronal reparative effects were observed (Patel et al., 2020; Jin et al., 2022). Such evidence demonstrates curcumin's potential to inhibit the NLRP3 inflammasome. It remains to be determined how curcumin modulates the NLRP3 pathway to safeguard neurons and minimize inflammatory response. Furthermore, the anti-oxidative, anti-apoptotic, and neuroprotective regulation of curcumin in NLRP3 inflammasome, especially in ischemic stroke, is yet to be explored. In this review, the PubMed, China National Knowledge Infrastructure (CNKI), and Web of Science databases were searched until to August 2023 using the keywords “curcumin,” “ischemic stroke” OR “stroke,” and “NLRP3” OR “NLRP3 inflammasome” to demonstrate the pathways network between NLRP3 and curcumin therapy in ischemic stroke.

## 2 The NLRP3 inflammasome and its function in ischemic stroke

### 2.1 Activated NLRP3 inflammasome after ischemic stroke

NLRP3 is a 115 kDa cytosolic protein and combines with caspase-1 and apoptotic-associated speck-like protein (ASC) to form the NLRP3 inflammatory complex. This complex can be activated by the innate immune system, including damage-associated molecular patterns (DAMPs), to initiate caspase-1 and IL-1β/IL-18 mutation and release and aggravate inflammatory reaction (Wang et al., 2022c). In the wake of ischemic stroke, increased DAMPs from injury cells and stimulated NLRP3 protein bind to the adapter protein ASC and pro-caspase-1, subsequently triggering the maturation of precursors IL-1β and IL-18 to induce neuroinflammation. Proinflammatory GSDMD works with the NLRP3 inflammasome to activate caspase-1 and cause pyroptotic cell death. This is exacerbated by intracellular Ca^2+^, high mitochondrial ROS production, and leukocyte recruitment that aggravate neuronal death (Yang et al., 2019). The NLRP3 inflammasome is expressed in immune cells such as neutrophils, dendritic cells, lymphocytes, epithelial cells, microglia, and neurons (Zahid et al., 2019). Both NLRP3 and NLRP3-dependent genes of the inflammasome are significantly elevated during the first phase of cerebral ischemia, intensifying cerebral ischemic injuries (Wang et al., 2022b). Interestingly, the suppression of NLRP3 has improved ischemic insult and neurovascular complications (Dodd et al., 2021).

#### 2.1.1 TLR4/NF-κB

The TLR4 is a pathogen recognition receptor. It is expressed mainly on microglia and, to a lesser extent, on astrocytes and neurons in both the central nervous system and ligands exogenous (PAMPs) or DAMPs that activate inflammation (Li et al., 2022a). TLR4 is stimulated by DAMPs in ischemic stroke, which then leads to the activation of NF-κB and AP1 via the MyD88-dependent signaling pathway. TLR4 signal activation causes the NLRP3 protein to trigger the NLRP3 inflammasome. This mechanism has been connected to NLRP3 dysfunction, including augmented release of pro-inflammatory cytokines (Luo et al., 2022b). The NF-κB transcription receives the signal from TLR4 and targets surface PPRs, leading to NLRP3 activation (Jin et al., 2019). Hence, TLR4/NF-κB is an upstream for NLRP3 to induce inflammation. TAK-242, a specific inhibitor of TLR4, inhibits NLRP3 inflammasome by curtailing NLRP3 and caspase-1 expression in oxygen–glucose deprivation reperfusion (OGD/R) BV2 cells (Liu et al., 2021a). Meisoindigo, an anti-inflammatory drug, has suppressed TLR4 and NF-κB proteins in ischemic stroke in a dose-dependent manner to alleviate brain damage. In the aforementioned study, meisoindigo prevented the alterations in the ischemic hemisphere 3 days after MCAO and OGD/R BV2 cells by downregulating the expression of TLR4/NF-κB, NLRP3, and M_1_ microglia. Meanwhile, the upregulation of NLRP3 and M_1_ microglia-related proteins could be improved by meisoindigo treatment in LPS-induced TLR4 activation (Ye et al., 2019). In addition, the inhibition of the TLR3/NF-κB pathway by Renshen Shouwu extract increased the newly developed neurons, thus improving neurological deficit after ischemic stroke while NLRP3 inflammasome expression was downregulated (Li et al., 2020c).

#### 2.1.2 Mitochondrial dysfunction

Mitochondrial dysfunction, including the overproduction of ROS, uncontrolled mitochondrial autophagy, and abnormal fission and fusion have been implicated in ischemic stroke (Yang et al., 2018; Lei et al., 2021; Shi et al., 2022b)**.** In addition, apoptosis, ATP disruption, calcium buildup, and faulty mitochondrial biogenesis contribute to aberrant ROS production during ischemic stroke (Bai et al., 2021). ROS release from mitochondria and mitochondrial DNA (mtDNA) damage leads to the activation of the NLRP3 inflammasome and NF-κB pathway (Li et al., 2021a). Furthermore, mitochondrial malfunction aggravates ROS over-production to further promote NLRP3 inflammasome activation. It is notable that ROS and mitochondrial function are connected and controlled by the NLRP3 inflammasome (Wang et al., 2019b). Idebenone, a mitochondrial protectant, has suppressed NLRP3 inflammation by mitigating mitochondrial dysfunction induced by cerebral ischemia/stroke and microglia overactivation to alleviate infarct volume and neurological deficit (Peng et al., 2020). Mitochondrial depolarization and mtDNA damage occur in OGD/R BV2 cells. Diazoxide reverses mtDNA damage and NLRP3 inflammasome assembly in primary microglia, indicating that mitochondrial dysfunction is fundamental to NLRP3 inflammasome activation (Gong et al., 2018). Furthermore, increased ROS and uncontrolled cell mitochondrial autophagy by a variety of molecular signaling pathways have been related to the stimulation of NLRP3 inflammasome (Zhou et al., 2011; Zeng et al., 2022). In the OGD/R PC12 cell, Taohong Siwu decoction, a traditional Chinese medicine, inhibits ROS and NLRP3 activation while upregulating mitophagy-related proteins including Parkin and PINK1. These were reversed by mitochondrial division inhibitory factor 1, indicating that mitophagy is a negative regulator of NLRP3 inflammasome activation (Shi et al., 2023a). Electroacupuncture also upregulates the expression of mitophagy-associated proteins while suppressing ROS-induced NLRP3 expression, leading to improvement in cognitive and neuronal impairments (Zhong et al., 2022). Furthermore, ketogenic diets inhibit ROS and TXNIP/NLRP3 inflammasome activation by suppressing mitochondrial fission and downregulating mitochondrial translocation-related proteins to improve middle cerebral artery occlusion/reperfusion (MCAO/R)-injury (Guo et al., 2018). Dynamin-related protein 1 (Drp1) is a key regulator of mitochondrial fission. The pharmacological inhibition of Drp1 translocation prevents mitochondrial fragmentation and protects neurons from oxygen–glucose deprivation (OGD)-induced injury (Zhao et al., 2013). The suppression of ER stress and/or ROS generation has also been shown to alleviate NLRP3-mediated inflammation in stroke cases by inhibiting Drp1-related mitochondrial function (Guo et al., 2018). Moreover, oxytocin reduces mitochondrial fission and oxidative stress within 3 days of intracerebral hemorrhage by downregulating NLRP3, ASC, and caspase-1 expressions and upregulating p-PKA and p-DRP1 expressions to alleviate neurological dysfunction (Yang et al., 2023b). These were revised by oxytocin or PKA inhibitors (Yang et al., 2023b), indicating that mitochondrial fission is related to the activation of NLRP3 inflammasome.

#### 2.1.3 Autophagy

The crosstalk between NLRP3 and autophagy reveals autophagy-regulated NLRP3 activation through the removal of ROS-producing damaged mitochondria and inflammasome cytokines (Saitoh and Akira, 2016; Biasizzo and Kopitar-Jerala, 2020). The inhibition of glycogen synthase kinase 3β (GSK-3β) could decrease NLRP3 inflammasome activation and upregulate LC3-II and p62 expression to reduce cerebral infarct volume, while 3-MA (autophagy inhibitor) could rescind the neuroprotective effect (Wang et al., 2019a). Autophagy intervention in an early stage of MCAO could ameliorate cerebral I/R injury through NLRP3-induced inflammation (Fu et al., 2022). NLRP3 inflammasome activation was impaired, and LC3-autophagy activation was increased, by 6-Gingerol, an anti-autophagic and anti-inflammatory medicinal agent. More importantly, this was reversed by the autophagy inhibitor 3-MA, implying that the inhibition of autophagy could increase NLRP3 expression and cell apoptosis (Luo et al., 2021). In addition, sinomenine inhibits NLRP3 and ASC expression in MACO mice and OGD/R cell models by inhibiting LC3-II-related autophagy and inflammation (Qiu et al., 2016). All these studies suggest that autophagy dysfunction has a significant impact on inflammation through NLRP3 activation. Autophagy inhibition could be significant in ameliorating inflammation in ischemic stroke. Conversely, autophagy activation in stroke may be beneficial for ischemic-induced neuronal injury. Geniposide in particular has been found to activate autophagy by increasing LC3 and beclin1 expressions and decreasing P62 expression while inhibiting the NLRP3 inflammasome (Fu et al., 2020). By preventing NLRP3 inflammasome activation-induced pyroptosis, moderate hypothermia therapy triggers autophagy to mitigate cerebral ischemic injury (Tu et al., 2019). Hence, autophagy may be advantageous or harmful, depending on the various stages of ischemic stroke and the interactions between signals and drug administration (Zhang et al., 2020). Silent information regulator family protein 1 (SIRT1) is essential for autophagy initiation and has been implicated as a regulator of autophagy in ischemic stroke (Teertam and Phanithi, 2022). It is notable that SIRT1 impairment after MCAO treatment activates the NLRP3 inflammasome and is reversed by SIRT1 inhibitors (i.e., EX527 and arctigenin) to exert neuroprotection by inhibiting SIRT1-dependent NLRP3 inflammasome (Zhang et al., 2017). Moreover, resveratrol (a SIRT1 agonist) has been demonstrated to inhibit MCAO-induced NLRP3 inflammasome activation by upregulating autophagy-related proteins including LC3B-II/LC3B-I and p62 to exert neuroprotection (He et al., 2017). The aforementioned studies indicate that SIRT1 is crucial for anti-NLRP3 inflammasome and regulating autophagy in ischemic stroke. Moreover, AMPK, an upstream of autophagy, has been linked to inflammation and NLRP3 expression in OGD/R and MCAO models (Huang et al., 2021). Aldolase A (ALDOA), the key protein in glycolysis flux and the mitochondrial damage process, is indispensable for NLRP3 activation. The inhibition of ALDOA regulated the clearance of damaged mitochondria depending on the AMPK activation and the SQSTM1/p62 transcription to control NLRP3 inflammasome activation (Bai et al., 2022). This could mean that ALDOA is a target of NLRP3 inflammasome activation through the AMPK–autophagy pathway (Bai et al., 2022). In ischemic stroke, the NLRP3-related protein expression that mitigates autophagy activation includes AMPK, mTOR, and ULK1. They have improved cognitive impairment and attenuated inflammation (Huang et al., 2021; Zhai et al., 2023). SINO, an anti-inflammatory drug, inhibits AMPK-mediated NLRP3 inflammasome activation against ischemic-induced brain injury (Qiu et al., 2016). In summary, autophagy regulators are potential targets for modulating NLRP3 inflammasome in ischemic stroke.

#### 2.1.4 Microglia receptors and phenotype transformation

NLRP3 inflammasome could be regulated and activated by microglia receptors and molecules, along with chemoattractant and chemokines after ischemic stroke (Franke et al., 2021). NLRP3 inflammasome activation is bound up with microglia phenotypes including proinflammatory M_1_ and anti-inflammatory M_2_ (Yang et al., 2023a). CX3CR1 is highly expressed in microglia. The inhibition of chemokine CX3CR1 improves neurologic function and microglia inflammation in ischemic stroke (Tang et al., 2014). Furthermore, exogenous rCX3CL1 had a neuroprotective effect in MCAO mice by decreasing NLRP3 inflammasome-induced pyroptosis and NF-κB expression (Ge et al., 2022). In addition, microglia M_1_ polarization in BV2 cells has upregulated both CX3CR1 and CX3CL1 expressions in chronic brain hypoperfusion rats (Mao et al., 2020). This was negated by miR-195 that mimics downregulated M_1_ phenotypic expression (Mao et al., 2020). This indicates that CX3CR1/CX3CL1 regulation may relate to microglia M_1_/M_2_ cytokine and NLRP3 inflammasome. One of the key proteins that trigger the NLRP3 inflammasome to release inflammatory factors is receptor-interacting protein kinase 1 (RIPK1). This can initiate necroptosis by activating RIPK3. It is notable that inhibited RIPK1 did reduce the volume of cerebral infarction (Deng et al., 2019). RhTrx-1, a RIPK1 inhibitor, can minimize neuronal injury by inhibiting NLRP3 activation, ROS-induced mitochondrial damage, and altering microglial M_1_/M_2_ phenotype (Jiao et al., 2020). The triggering receptor expressed on myeloid cells 1 (TREM-1) is a surface molecule on macrophages and microglia that increases pro-inflammatory mediator secretion and release in ischemic stroke. LP17, as the TREM-1 inhibitor, ameliorates neurological deficit and ischemic brain damage by decreasing NLRP3, caspase-1 and GSDMD mutation and release, and by decreasing microglia M_1_ expression (Liang et al., 2020a). The purinergic 2X7 receptor (P2X7R) has been reported to be activated with TLR4 and NLRP3 formation, as well as M_1_/M_2_ phenotypes in inflammatory disease (Ren et al., 2021). The inhibition of NLRP3 along with shifting microglia polarization toward the protective M_2_ phenotype by ketogenic diet regulates the activation of the P2X7R and TLR4/MyD88/NF-κB/NLRP3 pathways in multiple sclerosis (Sun et al., 2023). P2X7R was inhibited by dexmedetomidine (Dex) to reduce MCAO brain infarct size along with the downregulation of caspase-1 p10 expression (Sun et al., 2021). Several medications, including midazolam, d-Carvone, the inhibitor of the Takeda G-protein-receptor-5 (TGR5), INT777, and JLX001, could prevent and suppress cell pyroptosis to downregulate the expression of inflammatory factors and NLRP3-related proteins to protect cortical neurons from ischemic stroke (Dai et al., 2020; Bian et al., 2021; Liang et al., 2021; Shi et al., 2022a). In addition to medicinal intervention, moderate-intensity continuous exercise training deactivates NLRP1/NLRP3 and inhibits NF-κB p65 to switch M_1_ microglia to the anti-inflammatory M_2_ phenotype and promote functional recovery after ischemia/reperfusion (I/R) injury (Liu et al., 2022).

### 2.2 NLRP3 inhibition may mitigate neuronal pyroptosis and loss

Neuronal pyroptosis, a determining factor in neuronal death in cerebral ischemic-induced brain injury, is attenuated by NLRP3 inhibition. NLRP3 can also recruit apoptotic-associated proteins containing the caspase domains, which can then activate caspase-1 to cleave pro-IL-1β and pro-IL-18 trigger releases to initiate neuronal death (Feng et al., 2020). The administration of NLRP3 inhibitor MCC950 decreased infarct volumes to protect BBB integrity in ischemic stroke (Franke et al., 2021). The low-density lipoprotein receptor (LDLR) has been shown to be a regulator of NLRP3-induced neuronal pyroptosis. The NLRP3 inflammasome overactivation and long-term impairments in cognition and memory were found in LDLR-deficient MCAO mice (Sun et al., 2020a). NLRP3 inhibitor treatment in LDLR-deficient mice mitigated NLRP3-related neuroinflammation following ischemic stroke (Sun et al., 2020b). TLRs interact with NLRP3 to increase tissue inflammation and damage (Tajalli-Nezhad et al., 2019). The pathogenic-synergistic TLRs/NF-κB/NLRP3 pathway did cause excessive microglial activation and synaptic dysfunction. However, this was reversed by NLRP3 inhibitors to improve dopaminergic neuronal loss and motor deficit (Lee et al., 2019; Li et al., 2021c). In MCAO mice, intermittent theta-burst rTMS protected against neuronal damage and neurobehavior improvement by inhibiting the expression of neuronal pyroptosis-associated proteins, including caspase-1 and GSDMD and TLR4/NF-κB/NLRP3 signaling pathway in the peri-infarcted area (Luo et al., 2022b). Caspase-1 was also blocked by VX-765 to preserve the BBB integrity in the MCAO model and downregulate the pyroptotic protein expression, including NLRP3, GSDMD, and inflammatory-related factors, subsequently ameliorating ischemic-induced infarction and neuronal injury (Liang et al., 2020b; Franke et al., 2021). Moreover, the absence of IL-1β significantly reduced infarct volume and cell death in MCAO mice by downregulating IL-6, TNF-α, and pyroptotic-related molecule expression (Li et al., 2020a). These findings demonstrate that pyroptosis-related proteins may be a therapeutic target for ischemic stroke (Tuo et al., 2022).

### 2.3 microRNAs and stem cell therapy could inhibit NLRP3 in ischemic stroke

MicroRNAs are important post-transcriptional regulators and are involved in various neurological disorders, including ischemic stroke. As one of the important inflammation-regulated microRNAs, miR-139 targets c-Jun, a part of activation protein −1 (AP-1), and modulates NLRP3 activation to reduce inflammation in OGD/R BV-2 and SH-SY5Y cells (Wang-S. et al., 2020a). Furthermore, miR- 423-5p inhibitor could inhibit NLRP3 inflammasome activation to alleviate cerebral ischemic/reperfusion injury (Luo et al., 2022a). miR-203a-3p and miR-153-3p as AMPK upstream targets inhibit apoptosis and oxidative stress by regulating NLRP3 inflammasome activation (Li et al., 2022b). The upregulation of miR-139-5p contributes to NLRP3-induced pyroptosis via the downregulation of FOXO1 and TXNIP expression (Yao et al., 2022). MiR-668 inhibitors also impair NLRP3 inflammasome activation and inflammatory cytokine expression, resulting in a reduction in the infarct area of ischemic brains (He and Zhang, 2020). Moreover, long noncoding RNAs (lncRNAs) are endogenous regulatory RNA molecules that are essential regulators in cerebral ischemic injury. lncRNA NEAT1, as one key lncRNA, regulates miR-22-3p and participates in neuronal pyroptosis inhibition (Zhang et al., 2021). Gastrodin, from the Chinese herb Tianma, could inhibit NLRP3 inflammasome activation and antioxidant by regulating the lncRNA NEAT1/miR-22-3p axis (Zhang et al., 2021).

Based on decreased risk, reduced immunogenicity, and anti-inflammatory function, stem cell therapies have shown considerable clinical promise in the treatment of stroke. In particular, human cord blood-derived multipotent stem cell (HCB-SC) therapy minimizes brain infarct size, infarct volume, improves neurobehavioral functioning, and prolong stroke survival. Furthermore, lymphocytes co-cultured with HCB-SCs in tMCAO mice augment CD4 ^+^ CD25 ^+^ Foxp3 ^+^ Tregs in peripheral blood and reduce inflammation by suppressing NLRP3 inflammasome (Zhao et al., 2019). A cell graft with fresh human umbilical cord blood mononuclear cells has been used in MCAO rats to enhance neovascularization and inhibit NF-κB, NLRP3, cleaved caspase-1, and IL-1β expressions (Liu et al., 2018a). Furthermore, neural stem cell therapy has downregulated the expressions of TLR4- and NLRP3-related proteins in microglia to show neuroprotective and anti-inflammatory effects (Wang et al., 2023). Moreover, exosomes secreted from stem cells have exerted anti-inflammatory, angiogenesis, and neurogenesis effects by inhibiting NLRP3-induced neuronal pyroptosis including NLRP3, ACS, caspase-1, and mature IL-1β and IL-18 expression that were activated by MCAO (Liu et al., 2021b).

Table 1 summarizes the studies that evidence the beneficial effects that follow NLRP3 inhibition in cerebral ischemic stroke through various molecular pathways.

**TABLE 1:** NLRP3 inhibition by various interventions in ischemic stroke.

Drug name	Type of study	Treatment method		Treatment duration	Outcome	Targets or pathways	Reference
		Experiment group	Control group				
Sulforaphane; Genipin; MCC950	C57Bl/6 tMCAO mice model	Sulforaphane (25 mg/kg),	0.1% DMSO i.p. injection	Before occluding the MCA or after the 60 min of tMCAO	Reduced brain infarct volume and TUNNEL+ with NLRP3 neuron cell	Impaired NLRP3 activation	Franke et al. (2021)
		Genipin (2 mg/kg), MCC950 (50 mg/kg), i.p. injection					
CY-09	C57BL/6 and Ldlr^−/−^ MCAO mice	CY-09 (40 mg/kg) i.p. injection	Normal saline, i.p. injection	1 h before MCAO surgery	LDLR could suppress neuronal pyroptosis by inhibiting NLRP3 inflammasome activation	Inhibited NLRP3 activation	Sun et al. (2020a)
Ginsenoside Rd	C57BL/6 MCAO mice	Ginsenoside Rd (10 mg/kg, 20 mg/kg, and 40 mg/kg), i.p. injection	1,3-Propanediol, i.p. injection	30 min before MCAO/R and supplemented 2 h after MCAO/R	Decreased cerebral ischemia/reperfusion (I/R) injury by reducing neuronal pyroptosis	Suppressed ROS/TXNIP/NLRP3 inflammasome through the miR-139-5p-mediated FoxO1/Keap1/Nrf2 signaling pathway	Yao et al. (2022)
MCP	C57BL/6 MCAO mice	MCP (200 mg/kg/d, 400 mg/kg/d, and 800 mg/kg/d), i.p. injection	Sterile saline, i.p. injection	7 days before MCAO operation to 1 day after reperfusion	Mitigated neurological deficit scores, brain water content, and infarction volume	Impaired TLR4/NF-κB/NLRP3 inflammasome in microglia	Cai et al. (2023)
Idebenone	SD MCAO rats	Idebenone (100 mg/kg), i.p. injection	5% arabic gum saline solution	After reperfusion	Improved infarct volume and neurological deficit	Suppressed NLRP3 inflammation and mt-ROS	Peng et al. (2020)
YC-1	SD MCAO rats	YC-1 (5 mg/kg), i.p. injection	1% DMSO	2 h before MCAO	Alleviated NLRP3/caspase-1 and rescued immune cell infiltration	Inhibited HIF-1α and NLRP3/caspase-1	Jiang et al. (2020)
EA	SD MCAO rats	At the Shenting (DU24) and Baihui (DU20) acupoints	None	After 24 h of reperfusion, 30 min per day for 7 days	Attenuated cognitive and neuronal impairment	Suppressed ROS-induced NLRP3 expression	Zhong et al. (2022)
MiR-668 inhibitor	SD MCAO rats	miR-668 inhibitor,	miRNA control	10 min before reperfusion	Improved infarct volume and neurological deficit	Inhibited NLRP3, apoptosis, and mitochondrial function	He and Zhang (2020)
6-Gingerol	SD MCAO rats	6-Gingerol (3 or 6 mg/kg), i.p. injection	Normal saline + 1% dimethyl sulfoxide	30 min before MCAO	Against cerebral ischemia/reperfusion induced neuron injury	Inhibited NLRP3 inflammasome and apoptosis via TRPV1/FAF1-mediated autophagy	Luo et al. (2021)
SINO	C57BL/6 MCAO mice	SINO (10 or 20 mg/kg), i.p. injection	Normal saline	Daily for 3 days after MCAO (first injection administered 30 min after operation)	Alleviated cerebral injury after ischemic stroke	Suppressed NLRP3 inflammasome via AMPK signaling	Qiu et al. (2016)
Mild hypothermia treatment	SD MCAO rats	Environment kept at 4 C	Maintained at 25 °C	Start 2 h after pMCAO for 6 h	Alleviated diabetes-aggravated cerebral ischemic injury	Inhibited NLRP3 and autophagy	Tu et al. (2019)
Arctigenin	SD MCAO rats	Arctigenin (20 mg/kg), i.p. injection	Vehicle, i.p. injection	Per day before MCAO for 3 days	Attenuated ischemic stroke-induced neuroinflammation	Inhibited NLRP3 via the SIRT1 pathway	Zhang et al. (2017)
MiR-203a-3p and miR-153-3p	SD MCAO rats	MiR-203a-3p and miR-153-3p; 2 × 108 U/mL stereotaxic injection	miRNA control	Once; 3 days before surgery	Improved cognitive impairments	Inhibited NLRP3 via AMPK	Li et al. (2022a)
Luteolin	SD MCAO rats	Luteolin (10, 30, 60, and 90 mg/kg); ML385 (30 mg/kg); i.p. injection	0.1% DMSO/PBS solution	2 h after insults and then once daily until euthanasia	Improved neurologic function and reduced neuronal cell death	Inhibited NLRP3 via the Nrf-2 pathway	Zhang et al. (2021)
Ghrelin	ICR mice	Ghrelin (10, 20, or 30 μg), i.p. injection	Saline solution	1 h after ICH	Attenuated secondary brain injury post-ICH	Inhibited NLRP3 via the Nrf-2 pathway	Chen et al. (2020)
Formononetin	SD MCAO rats	Formononetin (30 mg/kg), i.p. injection	0.1% DMSO/PBS solution	Once daily for 3 days	Improved neurological function in MCAO rats	Inhibited NLRP3 and IL-1β and JAK2/STAT3 pathway	Yu et al. (2020)
Gastrodin	SD MCAO rats	i.p. injection	Normal saline	6 h prior to MCAO	Inhibited inflammation reaction	Inhibited p-STAT3 and NF-κB to downregulate NLRP3	Sun et al. (2021)
XQ-1H	C57BL/6 MCAO mice	XQ-1H, (62.4 mg/kg, 31.2 mg/kg, 15.6 mg/kg), i.p. injection	Normal saline	Once daily for 3 consecutive days after MCAO	Attenuated neuronal pyroptosis	Inhibited NLRP3/caspase-1 expression, IL-1beta/IL-18 mutation, and ROS release	Zhao et al. (2019)
Rabeprazole	SD MCAO rats	Rabeprazole (60 mg/kg), ethambutol (50 mg/kg), Pioglitazone (10 mg/kg), i.p. injection	Normal saline	Once before MCAO 30 min	Neuroprotective effects in the MCAO model	Inhibited NLRP3 via PPARγ	Ge et al. (2022)
Ethambutol							
Pioglitazone							
Exogenous rCX3CL1	C57BL/6 MCAO mice	1 or 2 μl exogenous rCX3CL1 (0.5μg/μl) by stereotaxic operation	PBS	1, 3, and 5 day(s) after reperfusion	Reduced neurological deficits and infarct lesion in mice after MCAO	Decreased NLRP3 inflammasome	Ge et al. (2022)
rhTrx-1	C57BL/6 MCAO mice	rhTrx-1 (10 mg/kg) by tail vein injection	0.9% sterile saline	Following reperfusion	Inhibited ischemic stroke-induced microglial neuroinflammation	Decreased NLRP3 inflammasome and switched microglia M1/M2	Jiao et al. (2020)
LP17	SD MCAO rats	LP17 (1 mg/kg) by intranasally	None	Once daily for 3 consecutive days after MCAO	Ameliorated neurological deficit scores and reduced ischemic brain damage	Impaired NLRP3 activation and decreased ROS.	Liang et al. (2020a)
Dexmedetomidine	SD MCAO rats	Dexmedetomidine (1 μg/kg) by tail vein at 0.05 μg/kg/min	0.1% DMSO	Beginning of operation and next 2 h after surgery	Reduced the MCAO brain infarct size	Inhibited NLRP3/caspase-1 pathway	Sun et al. (2021)
D-Carvone	Male Wistar MCAO	D-Carvone (10 mg/kg or 20 mg/kg, i.p. injection	0.1% DMSO	15 min before reperfusion and every day for 15 days	Contributed to cerebral stroke	Inhibited NLRP3 inflammasome activation and TLR3 pathway	Dai et al. (2020)
INT777	SD MCAO rats	INT777 (0.48 mg/kg) by intranasally	Normal saline	1 h after MCAO	Alleviated neuroinflammation after MCAO	Inhibited NLRP3 and cleaved-caspase-8 expression	Liang et al. (2021)
EET; MICET	C57BL/6 MCAO mice	EET	Standard condition group	3-day acclimation before operation	Rescued neurological deficits	NLRP1/NLRP3 de-activation to decreasing microglia inflammation	Liu et al., 2021a
		MICET					Liu et al. (2022)
miR-139	OGD/R BV-2 and SH-SY5Y cells	MiR-139 mimics	Mimics NC inhibitor NC	Before OGD/R	Upregulation of miR-139 exerted neuroprotection against OGD/R-induced nerve injury	NLRP3 inhibition reduced inflammation reaction	Wang et al. (2020a)
		MiR-139 inhibitor					
		NC					
GAS	SD MCAO rats	GAS (50 mg/kg)	Normal saline	7 days before I/R surgery and 7 days after surgery	Attenuated cerebral I/R injury	Inhibited NLRP3 via lncRNA NEAT1/miR-22-3p axis	Zhang et al. (2021)
Lymphocytes co-cultured with HCB-SCs	Male Wistar MCAO rats	Lymphocytes co-cultured with HCB-SCs (2×10^7^ cells)	Normal saline	Once at 2 h and 24 h after reperfusion	Exhibited a neuroprotective effect	Inhibition of NLRP3 and inflammatory factors	Zhao et al. (2019)
		By tail vein injection					
Exosome	SD MCAO rats	Exosome secreted from stem cell (80 μg, 100 μg, and 120 μg) by tail vein injection	PBS	2 h after reperfusion	Reduced brain infarct area	NLRP3 inhibition in neuron and microglia M2 phenotype	Liu et al. (2021b)
Renshen Shouwu extract	SD MCAO rats	Renshen Shouwu extract (50 mg/kg and 100 mg/kg), i.p. injection	0.5% CMC-Na	14 consecutive days after ischemic stroke	Enhanced neurogenesis and angiogenesis	Inhibited TLR4/NF-κB/NLRP3 signaling pathway	Li et al. (2020c)
Taohong Siwu decoction	OGD/R PC12 cell	Taohong Siwu -containing serum medium (5%, 10%, and 15%)	0.1% DMSO	24 h after OGD	Improved survival rate of OGD/R PC12 cells	Inhibited NLRP3 and upregulated mitophagy expression	Shi et al. (2023a)
Ketogenic diets	C57BL/6 MCAO mice	High-fat low-carbohydrate diet	Standard chow	3 weeks before MCAO	Improved brain ischemic tolerance	Inhibited Drp1 and NLRP3 and inflammasome activation	Guo et al. (2018)
Oxytocin	C57BL/6 ICH mice	Oxytocin (0.2 μg/g) intranasally	PBS	2 h, 1, 2, and 3 days after ICH	Improved neurological functions and alleviated neuronal pyroptosis and neuroinflammation	Decreased proinflammatory factors and alleviated OXTR/p-APK/DRP1 pathway	Yang et al. (2023b)

EA, electroacupuncture therapy; EET/MICET, enriched environment treatment and moderate-intensity continuous exercise training; HCB-SCs, human cord blood-derived stem cells; ICH, intracerebral hemorrhage; i.p., intraperitoneal; MCAO, middle cerebral artery occlusion; MCP, modified citrus pectin; OGD/R, oxygen–glucose deprivation/reoxygenation; PBS, physiologic saline; SD, Sprague–Dawley; SINO, sinomenine.

## 3 Neuroprotective functions and mechanisms of curcumin

Curcumin has been shown to be effective in improving pathological features and preventing the development of various diseases. It also modulates inflammatory and metabolic processes to protect cells from oxidative stress (Lin et al., 2022). This section elaborates on several pathways and factors that enhance cell synaptic plasticity, microglia phenotype, and gut microbiota involving curcumin.

### 3.1 Safeguarding neuronal synaptic plasticity

Curcumin has been shown to significantly enhance cognitive dysfunction and motor function in the central nervous system (Lo Cascio et al., 2021). Improvements in cognition are facilitated by reduced neuronal loss and hippocampal synaptic repair. The hippocampus is a significant part of the brain and involves several neuronal connections that control memory, learning, and emotional behavior (González-Granillo et al., 2022). A study showed that curcumin pre-treatment decreases neuronal death within the CA1 area of the hippocampi of LPS-induced depression rats, coinciding with improved synaptic function (Fan et al., 2021). In another study, curcumin inhibited long-term potentiation (LTP) in the synaptosomes of the hippocampal CA1 region and lowered Ca^2+^ concentration to improve synaptic plasticity (Shen et al., 2015). Additionally, curcumin reduced spontaneous neuronal activity, including changes in LFP phase coherence, to increase neuronal survival and the upregulation of synaptic proteins such as phospho-CaMKII and phospho-synapsin I in hippocampal slices exposed to Aβ-42 (Hoppe et al., 2013). In the dentate gyrus of the hippocampus, PSD thickness and synaptic width are increased by curcumin administration to enhance the number of newly developed neurons (Du et al., 2021). Furthermore, curcumin ameliorates synaptic mitochondrial dysfunction against age-related cognitive decline (Olesen et al., 2020). It also reduces synuclein formation and mitochondrial respiratory dysfunction in PD which has been exacerbated by ROS generated by chronic inflammation and severe motor and cognitive dysfunctions (Abdul-Latif et al., 2021). In cerebral ischemia/reperfusion, curcumin has minimized nerve damage and cognitive dysfunction, based on behavioral and immunohistochemical test results (Xu et al., 2019). In addition, the proliferation, differentiation, and maturation of neural stem cells were strengthened in the dentate gyrus area of the hippocampus after cerebral ischemia (Yang et al., 2021b). This followed the administration of curcumin.

### 3.2 Inhibiting ROS formation and microglia activation after ischemic stroke

ROS in astrocytes and microglia could be generated by nicotinamide adenine dinucleotide phosphate (NADPH) oxidase to drive the process of oxidative stress-induced brain damage after cerebral ischemia (Kahles et al., 2007; Orellana-Urzúa et al., 2020). Previous studies have demonstrated the anti-inflammatory and antioxidant properties of curcumin against neurodegeneration by its targeting of the NLRP3 inflammasome, PI3K/Akt/mTOR, and Nrf2 (Patel et al., 2020; Peng et al., 2021). By activating MAPK/Akt and PI3K/Akt signals, curcumin enhances the activity of antioxidant enzymes such as superoxide dismutase (SOD) and catalase against lipid and protein oxidation (Shah et al., 2007; Abrahams et al., 2019; Di Meo et al., 2019). Curcumin-induced Nrf2 upregulation in neurons curtailed ROS formation while increasing NO bioavailability (Shah et al., 2007). Heme oxygenase-1 (HO-1), a key protein, can interact with Nrf2 and be activated by curcumin to regulate inflammatory factors such as HIF-1α and NF-κB (Mhillaj et al., 2019). In OGD/R neurons and MCAO rats, curcumin attenuated OGD/R-induced NADPH upregulation to reduce oxidative stress by increasing the Akt/Nrf2 pathway to improve infarct size that could be reversed by Akt inhibitor (LY294002) (Wu et al., 2013).

Several studies have suggested that microglia activation may prevent chronic inflammation (Qin et al., 2019). Curcumin may alter microglia from M_1_ to M_2_, which then protects against neurological damage by blocking the ERK1/2 pathway and attenuating the formation of TNF-β, IL-1β, and IL-6 and NF-κB pathway activation in microglia (De Lorenzi et al., 2022). Microglia, as the immune cell in the brain, participates in the inflammation and release factors that determine the microglia phenotype (Ramírez-Carreto et al., 2023). Curcumin increased CD206 ^+^ Iba1 ^+^ microglia M_2_ phenotypic markers while decreasing M_1_ marker expression after 3 days in MCAO mice (Liu et al., 2017). Curcumin treatment also downregulated TLR4/NF-κB expression while upregulating TREM-2 expression, allowing the M_1_ pro-inflammatory phenotype (iNOS, IL-1, IL-6, and CD16/32) to be switched to the M_2_ anti-inflammatory phenotype (arginase 1, IL-4, IL-10, and CD206) (Zhang et al., 2019). By reducing pro-inflammation cytokines in subarachnoid hemorrhage, impaired TLR4 facilitates the decrement of brain water content and neurological deficits while curcumin treatment enhances these effects (Gao et al., 2019). Calmodulin-dependent protein kinase β (CaMKKβ) could activate the AMPK pathway. Its inhibition suppresses the effect of curcumin on LPS-induced BV2 microglia M_1_ phenotype gene expression (Qiao et al., 2020). It is noteworthy that interactions with neurons as well as changes in pathological stages and the release of numerous substances after brain injury may have an impact on curcumin-regulated microglia activation (Choi et al., 2011).

### 3.3 Regulating gut microbiota dysbiosis after ischemic stroke

Gut microbiota dysbiosis (GMD) is involved in the inflammatory response in neurodegenerative diseases such as depression and ischemic stroke (Fan and Pedersen, 2021). Curcumin acts on the microbiota–gut–brain axis to directly protect damaged neurons by regulating gut microbiota (GM) (Pluta et al., 2022). The gastrointestinal tract’s GM is composed of various bacterial species that regulate gut digestion and metabolism to maintain homeostasis (Di Meo et al., 2019). The GMD decreases intestinal barrier and BBB permeability, which may exacerbate brain neuroinflammation and edema and complicate post-stroke neuronal damage (Denes et al., 2011). In addition, the abundance of harmful substances, such as lipopolysaccharides released by abnormal bacteria, could activate microglia and astrocytes, leading to accumulated inflammatory cytokines that may worsen the tissue microenvironment (Jiang et al., 2017; Chen et al., 2019c). Curcumin alters microbiota composition, increases bacteria’s anti-inflammatory capacity, and decreases serum LPS levels in both PD and obesity (Wang et al., 2020b; Syeda et al., 2021). In a recent study, curcumin increased phosphatidylcholine in the prefrontal cortex and modulated specific gut microbiota, especially Muribaculaceae*,* which was key to alleviating anxiety-like behaviors (Zhang et al., 2022). Furthermore, curcumin improves the microbial richness, diversity, and composition of GM including Bacteroidaceae at the family level and *Prevotella* at the genus level—key bacterial species in AD development (Sun et al., 2020b). In ischemic stroke, gastrointestinal complications affect post-stroke outcomes in up to 50% of patients. In addition, intestinal microorganisms and neuroactive compounds from GM have an impact on the metabolism and immune condition that also affect neuronal behavior pattern after ischemic stroke (Pluta et al., 2021; Wang et al., 2022a). Ischemic stroke induces GMD with increased Enterobacteriaceae that exacerbate cerebral infarction (Yin et al., 2015). Moreover, curcumin balances both beneficial and harmful bacteria in the GM to reduce the growth of pathogens and the production of harmful substances (Di Meo et al., 2019). Short-chain fatty acids, which are metabolites of microflora, could reduce pro-inflammatory factors and NF-κB expression. In CIS, butyric acid, a short-chain fatty acid, improves GM, enriches the beneficial microbiota *Lactobacillus*, and repairs leaky gut to reduce neurological impairment (Chen et al., 2019b). Curcumin also restores short-chain fatty acid profiles and ameliorates intestinal barrier (Cai et al., 2023). Furthermore, it ameliorates intestinal epithelial barrier injury induced by oxidative stress by regulating Parkin-mitophagy and AMPK pathways (Cao et al., 2020). Curcumin reduces *Escherichia coli* growth and reduces gut inflammation by suppressing the TLR4/NF-κB (Gan et al., 2019). Thus, the regulation of curcumin in the GM of ischemic stroke is closely associated with the inflammatory pathways.

In summary, several pathways are employed by curcumin to minimize inflammation. In addition, curcumin modulates microglia polarization and GM. These help curtail neuronal loss.

## 4 Curcumin exerts neuroprotection in ischemic stroke by suppressing the NLRP3 inflammasome

As the vital active ingredient in turmeric, curcumin has beneficial pharmacological functions such as anti-inflammation, antioxidation, and neuroprotection in brain injury (Fan and Lei, 2022). The NLRP3 inflammasome inhibition of curcumin has the potential to restrict inflammation and restore injured neurons (He et al., 2018). Curcumin can control NLRP3 inflammasome activation that may be mediated by the downregulation of inflammatory pathways, reduction of cell ROS, or restoration of mitochondrial function (Patel et al., 2020). New therapeutic approaches and direct clinical translational studies for ischemic stroke and other diseases through curcumin's inhibition of NLRP3 and NLRP3-related inflammatory pathways according to recent investigations are illustrated in Table 2.

**TABLE 2:** Impairment of NLRP3 activation by curcumin.

Disease	Species	Treatment method	Outcome	Targets or pathways	Reference
MSU-induced peritonitis	NLRP3 knockout mice	Curcumin (100 mg/kg), i.p. injection	Suppressed inflammation	Prevented binding of ASC adapter to NLRP3 and suppressed NF-κB activation	Yin et al. (2018)
Gouty arthritis	C57BL/6; THP-1 and murine RAW264.7 macrophages	Curcumin (1 μM, 5 μM, and 10 μM) for cells; curcumin (150 mg/kg) by intraperitoneal injection	Improved mitochondrial function and reduced ROS release	Inhibited NLRP3 inflammasome and inflammatory expression	Chen et al. (2019a)
Lung inflammation	C57BL/6 mice	*Curcumin phaeocaulis* or MCC950 by intratracheal instillation	Antioxidation and NF-κB inhibition	Inhibited NLRP3 inflammasome	Nam et al. (2022)
DOX-induced cardiotoxicity	KM mice	Curcumin (50, 100, 200, and 400 mg/kg) by gavage	Attenuated cardiac function	Suppressed NLRP3 pyroptosis	Yu et al. (2020)
Renal interstitial fibrosis	Sprague–Dawley unilateral ureteral obstruction rats	Curcumin (200 mg/kg) by gastrogavage	Inhibited inflammation	Hindered NLRP3 via activating autophagy and mitochondria function	Luo et al. (2021)
TNFα-induced inflammation	Caco-2 cell monolayers	Curcumin (2–8 µM)	Mitigated inflammation	Repressed TNF-α and NLRP3 inflammasome	Iglesias et al. (2022)
LPS treatment	THP-1 cells	Demethoxycurcumin (10 μM); PPARγ antagonist T0070907 (10 μM)	Inhibited NLRP3 and NF-KB activation	Downregulated PPARγ expression	Tang et al. (2014)
Age-induced tight junction impairment	Sertoli cells	Curcumin (5, 10, and 20 Μm)	Increased mitochondrial activation and AMP/ATP metabolism	Inhibited NLRP3 and activated SIRT3/AMPT/SOD2 pathways	Xie et al. (2011)
Cerebral ischemic stroke	Male ICR mice and SD rat MCAO model	Curcumin (50 mg/kg), i.p. injection	Decreased brain infract area	Suppressed TXNIP-NLRP3 inflammasome active through AMPK-dependent manner	Li et al. (2015)
Cerebral ischemic stroke	C57BL/6 MCAO mice	Curcumin (150 mg/kg), i.p. injection	Reduced brain infract area	inhibited NLRP3 pyroptosis, NF-κB, and microglia polarization	Ran et al. (2021)
Lidocaine-induced cytotoxicity	PC12 cells	Curcumin (0, 2.5, 5, 10, 20, and 40 μM) and NLRP3 inhibitor MCC950 (5 μM)	Decreased cell death and apoptosis	Inhibited NLRP3	Li et al. (2020a)
Epilepsy	Sprague–Dawley rats	Curcumin (100 mg/kg) by oral gavage	Reduced neuronal loss	Impaired NLRP3 inflammasome	He et al. (2018)
Alzheimer’s disease	APP/PS1 transgenic mice	Curcumin nanomaterial (25 mg/kg) by caudal vein	Decreased β-amyloid plaque to restoring blood–brain barrier and memory deficits	Inhibited NLRP3 activation	Ruan et al. (2022)
Chronic Gulf War illness	Sprague–Dawley rats	Nanoparticle-encapsulated curcumin (10 or 20 mg/kg) by oral gavage	Ameliorated cognitive dysfunction	Hindered NF-κB/NLRP3 activation in microglia	Attaluri et al. (2022)
Diabetic peripheral neuropathy	Sprague–Dawley rats	Curcumin (20 or 40 mg/kg) by oral gavage	Decreased TUNEL-positive cells	Downregulated NLRP3 expression	Dwivedi et al. (2022)
Sepsis	ICR male mice	RGD-loaded curcumin liposomes by intravenous route	Inhibited inflammation	Curcumin downregulated NLRP3, cleaved-caspase-1, and IL-1β expression	Shi et al. (2023a)
Diabetic kidney disease	Sprague–Dawley rats	PLGA-GA2 nanoparticulate curcumin (20 or 40 mg/kg) by oral gavage	Suppressed inflammation	Inhibited P38 (MAPK) and P53 deactivation to decreasing NLRP3 inflammasome activation	Ganugula et al. (2023)
Cerebral ischemic stroke	Adult male Wistar rats BCCAO model	Triblock copolymer nanomicelles loaded with curcumin (40 and 80 mg/kg) by gavage	Impaired inflammation	Inhibited pNF-κB and inflammatory cytokines (TNF-α and IL-1β) expression	Li et al. (2021b)

BCCAO, bilateral common carotid artery occlusion; DAB, 1,2-diacetylbenzene; DOX, doxorubicin; MSU, monosodium urate crystal; NP, polymer-based nanoparticles; RGD, arginine–glycine–aspartic acid peptide.

Curcumin specifically impairs NLRP3 inflammasome activation without affecting either NLRC4 or AIM2 inflammasomes in LPS-induced bone marrow-derived macrophages (Yin et al., 2018). Moreover, in an LPS-induced disease, curcumin attenuates IL-1β secretion and regulates autophagy, sirtuin-2, and ROS to inhibit inflammation (Yin et al., 2018). In particular, curcumin suppresses DAB-induced TREM-1 and NLRP3 activation to alleviate cognitive impairment triggered by TLR4 and NF-κB upregulation (Nguyen et al., 2022). AI44 as a curcumin analog activates caspase-1 mutation, IL-1β, and pro–IL-18 by binding to peroxiredoxin 1 (PRDX1) but not to the TLR4/NF-κB pathway (Liu et al., 2018b). In THP-1 and murine RAW264.7 macrophages, monosodium urate induces NLRP3 inflammasome and NF-κB signaling upregulation that is reversed by curcumin, improving mitochondrial function to reduce ROS release and downregulate pro-inflammatory factors like Ik-Bα, IL-1β, and IL-6 (Chen et al., 2019a). Demethoxycurcumin prevents nanoparticle-induced NLRP3 activation in macrophages by inhibiting NF-κB (Nam et al., 2022). Moreover, curcumin decreases TNF-induced oxidant formation and NLRP3 inflammasome by downregulating the NF-κB, ERK1/2, and JNK pathways (Iglesias et al., 2022).

In neurological disease, neuronal injury and chronic stress could aggravate the inflammation accompanied by upregulated NLRP3 inflammasome (O'Brien et al., 2020). Curcumin has been shown to improve neuronal injury by regulating the NLRP3-related pathway and NLRP3-related inflammation (Ran et al., 2021). In depression, NF-κB and pro-inflammatory cytokine upregulation could be reversed by curcumin by decreasing IL-1β, TNF-α, and NLRP3 inflammasome expression (Ramaholimihaso et al., 2020). In ischemic-induced brain injury, NLRP3 inhibition through curcumin administration has been confirmed in several studies (Palomino-Antolin et al., 2022). MCC950 administration in NLRP3 knock-down mice increased ZO-1 and claudin-5 protein expression and decreased CCL-2/IL-1β expression to protect the BBB, improve neurological outcomes, and decrease infarct volume (Palomino-Antolin et al., 2022). In addition, α5β1 integrin expressed in endothelial cells showed that its knock-out could reduce BBB stabilization and significantly lower mitochondrial-induced oxidative stress and NLRP3 inflammasome, as well as claudin-5 and ZO-1 expression (Amruta and Bix, 2021). Curcumin protected the BBB stability of brain microvascular endothelial cells from OGD-induced permeability disruption by increasing HO-1 expression (Wang et al., 2013). Excessive glutamate is released after ischemic stroke along with ER and oxidative stresses. Curcumin regulates AMPK activity to rescue energy metabolic dysfunction and ER stress levels. Furthermore, curcumin protects hippocampal neurons from glutamate neurotoxicity by inhibiting TXNIP-NLRP3 inflammasome activity in an AMPK-dependent manner (Li et al., 2015). Moreover, NLRP3 activation in PC12 cells was inhibited by curcumin and NLRP3 inhibitor, MCC950, to decrease cell death and apoptosis (Li et al., 2020b). Curcumin has been shown to reduce neuroinflammation following ischemic stroke (Ran et al., 2021). It has also been observed to reduce pyroptotic proteins such as cleaved-caspase-1, GSDMS-N, and IL-1β following ischemic stroke (Ran et al., 2021). Additionally, curcumin has been shown to hinder the binding and activation of the NLRP3 inflammasome by inhibiting K^+^ and Ca^2+^ efflux, downregulating NF-κB, and diminishing the association between the ASC and NLRP3 (Yin et al., 2018; Hasanzadeh et al., 2020). Previous investigations have shown that curcumin and some of its analogs modulate magnesium ions to disrupt LPS-induced TLR4/MD activation (Zusso et al., 2019).

Autophagy and NLRP3 inflammasome have been identified as key players in cerebral I/R injury. Curcumin reduces early brain injury and improves neurological outcomes by suppressing NLRP3 inflammasome after ischemic stroke (Ran et al., 2021). Decreased LC3-II and HIF-1α expressions and increased p62 autophagy-related protein expression were observed following curcumin administration after an ischemic stroke (Forouzanfar et al., 2020). This implies that curcumin as an NLRP3 inflammasome inhibitor can regulate autophagy. More importantly, autophagy mediators could regulate NLRP3 inflammasome activation following curcumin administration in the wake of ischemic stroke. HIF-1α may be an NLRP3 inflammasome regulator in curcumin administration during stroke cases. A previous study showed both HIF-1α and NLRP3 to be upregulated after 6 h of brain ischemic reperfusion, continuing for 24 h (Jiang et al., 2020). A HIF-1α inhibitor (YC-1) significantly reduced NLRP3 inflammasome, resulting in the downregulation of cell apoptosis and pyroptosis (Jiang et al., 2020). Curcumin can also inhibit HIF-1α to alleviate brain injury by decreasing inflammation and TLR4/NF-κB/TNF-α and NO expression (Safdari et al., 2021). It can thus potentially regulate HIF-1α expression to influence NLRP3 and autophagy in ischemic stroke. Nonetheless, studies relating to curcumin in HIF-1α and autophagy are needed. The elevation of p-Akt/p-mTOR and the reduction of IL-1β, TLR4, p-38, and p-p38 levels have also been observed after curcumin administration in ischemic stroke and were concomitant with curtailed LC-3-II and NLRP3 markers (Huang et al., 2018). The PI3K/AKT/mTOR pathway plays a critical neuro-regulatory function through autophagy to support essential cellular function (Yang et al., 2021a). Curcumin ameliorates ischemic-induced brain impairment by upregulating the PI3K/AKT/mTOR pathway and downregulating autophagy and the TLR4/NF-κB pathway. However, the PI3K/AKT/mTOR (LY294002) or TLR4 (anisomycin) inhibitors suppressed these protective effects (Huang et al., 2018). Furthermore, curcumin inhibited NLRP3-related cell pyroptosis through autophagy, which was revised by the autophagy activator rapamycin (Yu et al., 2020). Autophagy is an important target of curcumin to regulate NLRP3 pathway. The PI3K/AKT/mTOR pathway interacts with autophagy and TLR4/NF-κB/NLRP3 pathways and could be indispensable to the anti-inflammatory and neuroprotective effects of curcumin. However, its effect on autophagy in NLRP3-driven neuroinflammation in ischemic stroke has not been evaluated.

Curcumin at 200 mg/kg and 300 mg/kg at 4 h post-MCAO significantly reduces the infarct area (Dohare et al., 2008). Curcumin crossed the BBB to exert maximum effect within 1 h and returned to normal levels in 2 days (Wang et al., 2005). It should be noted that curcumin has not been the subject of clinical trials due to its poor solubility and low absorption (Pan-On et al., 2022). Recently, better delivery systems such as polyester-based, nanomaterial, and arginine–glycine–aspartic acid (RGD)-loaded curcumin have attempted to improve its absorption and permeability. Notably, PLGA nanoparticles loaded with curcumin significantly enhance the oral absorption of curcumin (Xie et al., 2011). Additionally, curcumin-based nanotherapeutics inhibit NLRP3 inflammasome activation (Ruan et al., 2022). Furthermore, curcumin nanomaterial significantly improves memory deficit and the BBB to protect neurons from β-amyloid-induced activation of the NLRP3 related neuroinflammatory pathway (Ruan et al., 2022). Oral polymer nanoparticle-encapsulated curcumin has significantly enhanced synaptophysin puncta in the hippocampus of patients with chronic Gulf War illness to improve cognitive dysfunction by reducing ROS release and NF-κB/NLRP3 activation in microglia (Attaluri et al., 2022). Moreover, nanosystems containing curcumin (nCUR) have significantly decreased TUNEL-positive cells and mRNA expression of NLRP3, IL-1β, and macrophage infiltration to protect neurons from diabetic peripheral neuropathy (Dwivedi et al., 2022). In RGD-loaded curcumin liposome treatment, NLRP3, cleaved-caspase-1, and IL-1β remarkably decreased in LPS-induced macrophages (Shi et al., 2023b). PLGA-GA2 coupling nanoparticulate curcumin has good bioavailability and safety and has been demonstrated to exert anti-inflammatory effects in the livers and kidneys of diabetic kidney disease mice by inhibiting the P38 (MAPK) and P53 deactivation to decrease NLRP3 inflammasome (Ganugula et al., 2023). In ischemic stroke, curcumin nanoparticles have had better neuroprotective effects by downregulating NF-κB and pro-inflammatory expressions compared to using curcumin alone (Li et al., 2021b). Therefore, in ischemic stroke, investigations relating to cur–nanoparticles affecting NLRP3 inflammasome activation are warranted.

## 5 Conclusion and perspectives

**FIGURE 1:**
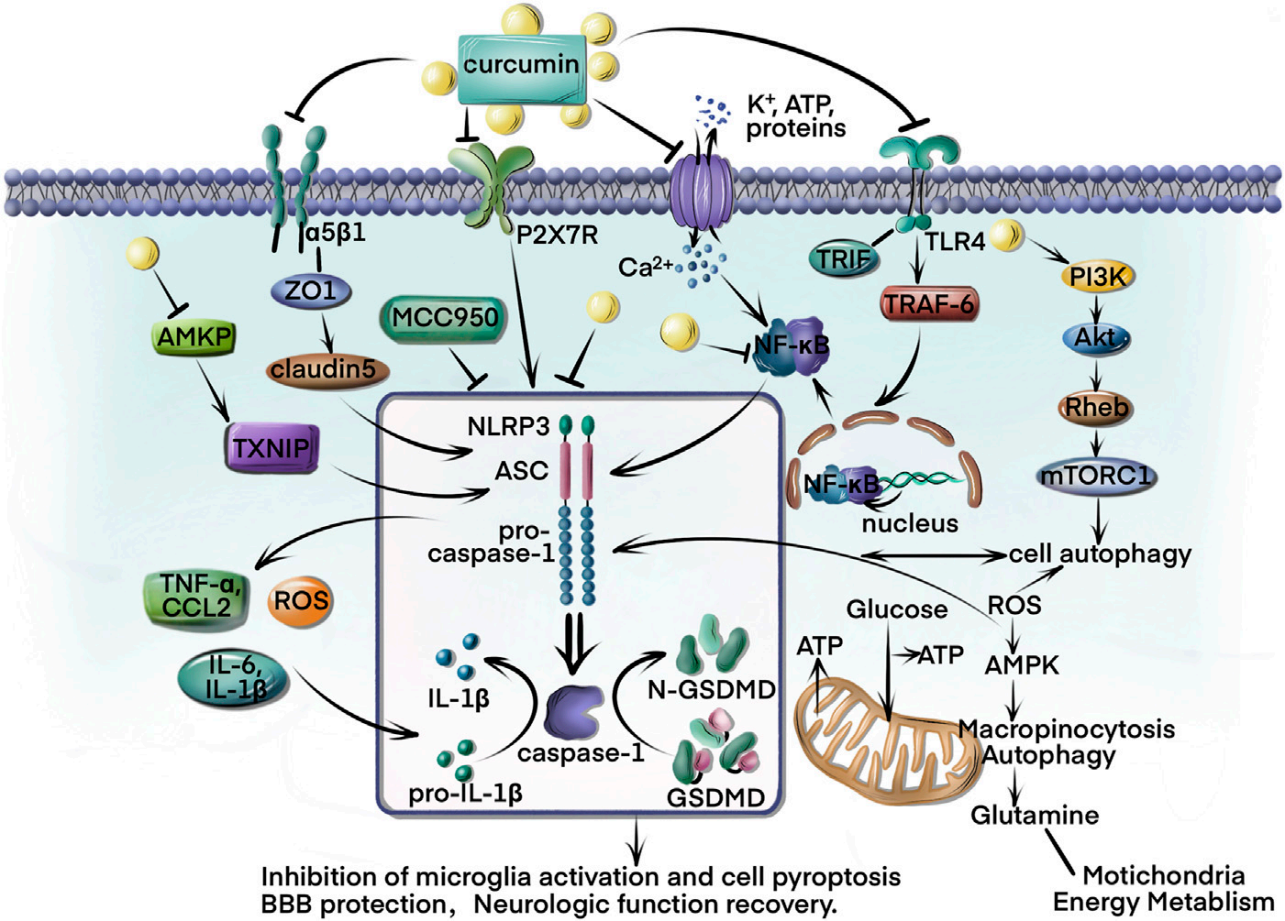
Curcumin is the primary component of turmeric’s rhizome and is currently under investigation for several clinical diseases. This review summarizes curcumin’s direct and indirect inhibition of NLRP3 inflammation activation in ischemic stroke. First, curcumin targets the NLRP3 to reduce caspase-1 and GSDMD cleavage, as well as IL-1β/IL-18 release and cell pyroptosis inhibition. Curcumin also regulates the imbalance of Ca^2+^ and K^+^ induced by ischemic stroke. In addition, curcumin modulates the AMPK/TXNIP/NLRP3 and P2x7R/NLRP3 pathways to rescue energy metabolism and reduce ROS formation. The BBB protection of curcumin has been demonstrated through regulation of ZO-1/claudin-5/NLRP3 pathways to downregulate TNF-α, IL-6, IL-1β, and CCL2 expressions. Furthermore, the TLR4/NF-κB/NLRP3 and autophagy-NLRP3 pathways play an important role in microglia alteration and inflammatory inhibition.

The inhibition of NLRP3 inflammasome is a new therapeutic approach to ischemic stroke for protecting injured neurons. By controlling effector molecules in the brain, curcumin, a strong inhibitor of NLRP3 inflammasome, has been proven to protect neurons from damage caused by ischemic stroke and neurological conditions. In this review, we evaluated how NLRP3 is activated by several pathways after ischemic stroke. Curcumin as a neuroprotective drug inhibits ROS formation and regulates microglia M_1_/M_2_ and gut microbiota to mitigate inflammation. The upstream and downstream pathways were clarified to show how curcumin regulates NLRP3 inflammasome to affect neuroinflammation and BBB integrity following ischemic stroke. The TLR4/NF-κB autophagy-related mediators and cell energy metabolism pathways were also clarified and the precise mechanisms shown by which curcumin affects the NLRP3 inflammasome (Figure 1). In conclusion, curcumin slows the development of ischemic stroke.

However, there are several challenges or limitations concerning the employment of curcumin. For instance, several mechanisms regarding the role of autophagy and NLRP3 in curcumin treatment after ischemic stroke remain to be investigated. Furthermore, specific mechanisms and targets of curcumin based NLRP3 inhibitors or NLRP3 knockout mice need to be explored in ischemic stroke treatment. Moreover, the effect of curcumin on gut microbiota in different stages after ischemic stroke along with the specific mechanisms involved needs to be assessed. This could facilitate understanding of the anti-inflammatory and protective effects of curcumin through the brain–gut axis. There are increasing reports relating to the protective effects of delivery systems-loaded curcumin, including RGD-load curcumin, PLGA-curcumin, and nano-curcumin. However, the bio-absorbability of these delivery systems-loaded curcumins are very different in ischemic stroke treatment. Therefore, more studies are needed to explore the recommended dose and therapeutic effect following the administration of curcumin, particularly nano-curcumin, for ischemic stroke in both preclinical and clinical settings. Meanwhile, to overcome the pharmacological limitations due to poor bioavailability, more analogs and derivatives of curcumin need to be developed to achieve high bioavailable and low toxic effects in future studies.

## Figures and Tables

**Table:** 

**ALDOA**	aldolase A
**AD**	Alzheimer’s disease
**ASC**	apoptosis-associated speck-like protein
**RGD**	arginine–glycine–aspartic acid peptide
**BBB**	blood–brain barrier
**CaMKKβ**	calmodulin-dependent protein kinase β
**CIS**	cerebral ischemia/stroke
**CKD**	chronic kidney disease
**DAMPs**	damage-associated molecular patterns
**Drp1**	dynamin-related protein 1
**DSS**	dextran sulfate sodium
**GMD**	gut microbiota dysbiosis
**HO-1**	heme oxygenase-1
**HCB-SC**	human cord blood-derived multipotent stem cell
**HIF1α**	hypoxia-inducible factor 1α
**iNOS**	inducible nitric oxide synthase
**I/R**	ischemia/reperfusion
**LFP**	local field potential
**LTP**	Long-term potentiation
**lncRNAs**	long noncoding RNA
**Ldlr**	low-density lipoprotein receptor
**MCAO**	middle cerebral artery occlusion
**nCUR**	nanosystems containing curcumin
**NLRP3**	NOD-like receptor thermal protein domain-associated protein 3
**OGD/R**	oxygen–glucose deprivation/reperfusion
**Nrf2**	nuclear factor erythroid 2-related factor 2
**PD**	Parkinson’s disease
**PRDX1**	peroxiredoxin 1
**PPAR**	peroxisome proliferator-activated receptor gamma
**PAF**	platelet-activating factor
**P2X7R**	purinergic 2X7 receptor
**RIPK1**	receptor‐interacting protein kinase 1
**ROS**	reactive oxygen species
**SIRT1**	silent information regulator family protein 1
**SOD**	superoxide dismutase
**TGR5**	Takeda-G-protein-receptor-5
**TLR4**	toll-like receptor 4
**TNF-α**	tumor necrosis factor-α
**TREM-1**	triggering receptor expressed on myeloid cells 1
